# Labeling and *in vivo* visualization of transplanted adipose tissue-derived stem cells with safe cadmium-free aqueous ZnS coating of ZnS-AgInS_2_ nanoparticles

**DOI:** 10.1038/srep40047

**Published:** 2017-01-06

**Authors:** Yusuke Ogihara, Hiroshi Yukawa, Tatsuya Kameyama, Hiroyasu Nishi, Daisuke Onoshima, Tetsuya Ishikawa, Tsukasa Torimoto, Yoshinobu Baba

**Affiliations:** 1 Department of Applied Chemistry, Graduate School of Engineering, Nagoya University, Furo-cho, Chikusa-ku, Nagoya 464-8603, Japan; 2 ImPACT Research Center for Advanced Nanobiodevices, Nagoya University, Furo-cho, Chikusa-ku, Nagoya 464-8603, Japan; 3 Department of Crystalline Materials Science, Graduate School of Engineering, Nagoya University, Furo-cho, Chikusa-ku, Nagoya 464-8603, Japan; 4 Institute of Innovation for Future Society, Nagoya University, Furo-cho, Chikusa-ku, Nagoya 464-8603, Japan; 5 Department of Medical Technology, Nagoya University, Graduate School of Medicine, Daikominami, Higashi-ku, Nagoya 461-8673, Japan; 6 Health Research Institute, National Institute of Advanced Industrial Science and Technology (AIST), 2217-14, Hayashi-cho, Takamatsu 761-0395, Japan

## Abstract

The facile synthesis of ZnS-AgInS_2_ (ZAIS) as cadmium-free QDs and their application, mainly in solar cells, has been reported by our groups. In the present study, we investigated the safety and the usefulness for labeling and *in vivo* imaging of a newly synthesized aqueous ZnS-coated ZAIS (ZnS-ZAIS) carboxylated nanoparticles (ZZC) to stem cells. ZZC shows the strong fluorescence in aqueous solutions such as PBS and cell culture medium, and a complex of ZZC and octa-arginine (R8) peptides (R8-ZZC) can achieve the highly efficient labeling of adipose tissue-derived stem cells (ASCs). The cytotoxicity of R8-ZZC to ASCs was found to be extremely low in comparison to that of CdSe-based QDs, and R8-ZZC was confirmed to have no influence on the proliferation rate or the differentiation ability of ASCs. Moreover, R8-ZZC was not found to induce the production of major inflammatory cytokines (TNF-α, IFN-γ, IL-12p70, IL-6 and MCP-1) in ASCs. Transplanted R8-ZZC-labeled ASCs could be quantitatively detected in the lungs and liver mainly using an *in vivo* imaging system. In addition, high-speed multiphoton confocal laser microscopy revealed the presence of aggregates of transplanted ASCs at many sites in the lungs, whereas individual ASCs were found to have accumulated in the liver.

Stem cell therapy plays an important role in regenerative medicine, especially for tissues and organs that are difficult to reconstruct due to their complicated structures and functions such as the guts, liver and islets^1^,^2^,^3^. Numerous types of somatic stem cells have been established thus leading to numerous medical applications in regenerative medicine, because their stem cells have been already confirmed to be safe in comparison to embryonic stem (ES) cells and induce induced pluripotent stem (iPS) cells^4^,^5^,^6^,^7^. Adipose tissue-derived stem cells (ASCs) have received a great deal of attention in recent years as multipotent somatic stem cells. Large amount of ASCs can be obtained using methods that are relatively easy to perform such as lipoaspiration under local anesthesia. These cells have the ability to self-renew and to efficiently differentiate in various types of mesenchymal cells, including adipocytes and osteocytes^8^,^9^. The development of technologies to label and image ASCs has therefore become important in making further progress in their clinical applications.

Quantum dots (QDs), which are known as semiconductor nanoparticles, are inorganic fluorescence probes that have many distinctive advantages in comparison to organic or protein probes, including high luminance, superior photostability, high quantum yields and wide excitation wavelengths^10^,^11^. Based on these characteristics, QDs have recently received a great deal of attention as fluorescence probes for biomolecules and live cells, including stem cells^12^. Our group has developed QD labeling methods for stem cells using cationic liposomes and octa-arginine (R8) peptides (also known as cell-penetrating peptides)^13^,^14^.

However, well-developed QDs contain toxic elements, including (but not limited to) Cd, Pb, Hg, Se and Te. Thus, the development of novel QDs with extremely low cytotoxicity (especially Cd-free alternatives) is necessary for further advancement of QD technology^15^,^16^,^17^,^18^. There have been some reports about the cadmium-free QDs such as carbon or CuInS_2_/ZnS QDs^19^,^20^,^21^. We previously reported the facile synthesis of ZnS-AgInS_2_ (ZAIS) as a cadmium-free QD, and confirmed that ZAIS was useful as a color-adjustable fluorophore mainly for solar cells^22^,^23^. ZAIS exhibited intense fluorescence from about 500 nm to 800 nm (which includes near-infrared region) at room temperature, when the composition rate of AgIn and Zn were changed in the ZAIS nanoparticles. At present the biological application of ZAIS, especially stem cell labeling and *in vivo* imaging remains largely uninvestigated^24^,^25^,^26^,^27^.

In the present study, ZnS-coated ZAIS nanoparticles (ZnS-ZAIS) was carboxylated in order to allow for its dispersal in aqueous solutions such as phosphate buffered saline (PBS) and cell culture medium. Then, the fluorescence labeling of ASCs by ZZC and R8 complex (R8-ZZC) was then addressed. The influence of R8-ZZC labeling on the properties of ASC, especially in regard to the induction of major inflammatory cytokines, was investigated, and *in vivo* fluorescence imaging of transplanted R8-ZZC-labeled ASCs was performed in mice. We also investigated the accumulation of transplanted ASCs using high-speed multiphoton confocal laser microscopy.

## Results

### The properties of ZZC and R8-ZZC

**Figure 1:**
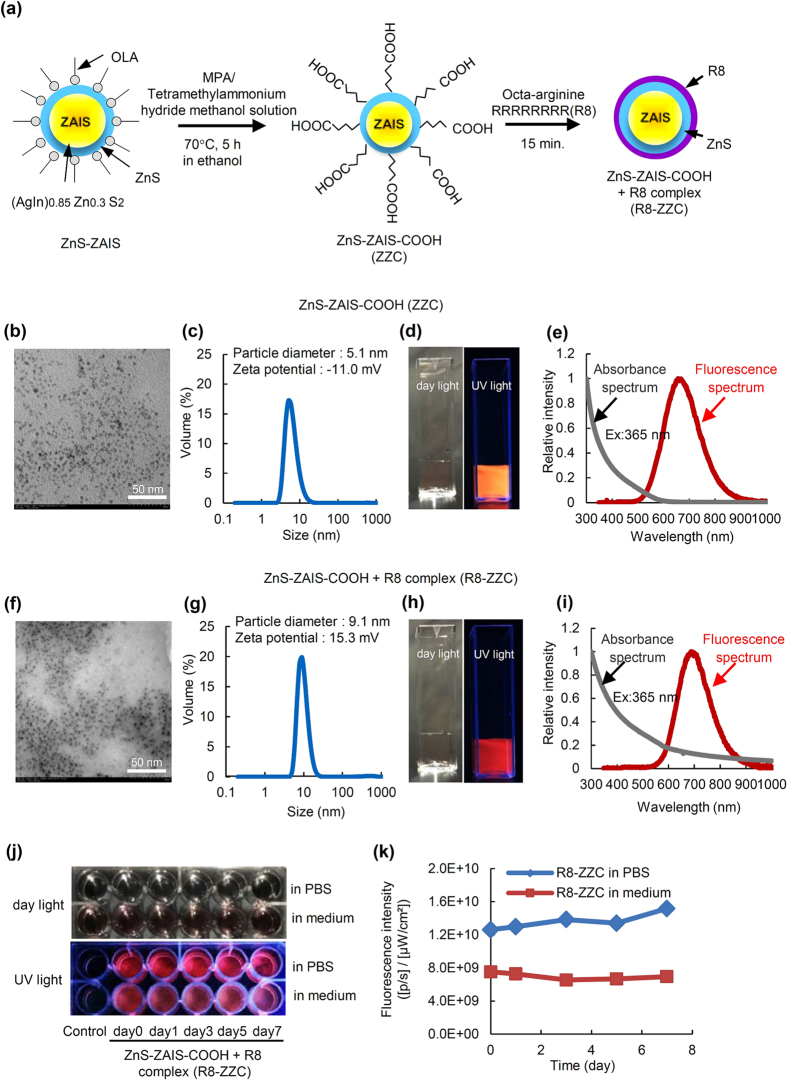
The synthesis protocol and the fluorescence properties of ZZC and R8-ZZC. (**a**) A schematic diagram of the synthesis of ZZC and R8-ZZC. (**b**) An SEM image, (**c**) the particle diameter and zeta potential, (**d**) fluorescence images (excited by day light and UV light), and (**e**) the absorbance and fluorescence spectrum of ZZC in water. (**f**) An SEM image, (**g**) the particle diameter and zeta potential, (**h**) fluorescence images (excited by day light and UV light), and (**i**) the absorbance and fluorescence spectrum of R8-ZZC in water. (**j**) The fluorescence images and (**k**) intensitiesof R8-ZZC (8 nM of ZZC) in PBS or the culture medium of ASCs after 7 days of culturing (37 °C, 5% CO_2_).

The synthesis scheme of ZZC and R8-ZZC is shown in Fig. 1(a). TEM and fluorescence images and the particle size distribution, the zeta potential, absorbance and the fluorescence spectra of ZZC and R8-ZZC are shown in Fig. 1(b–i). The ZZC and R8-ZZC particles were observed to be smaller than 10 nm in size (Fig. 1(b,f)). The particle sizes of ZZC and R8-ZZC were 5.1 nm and 9.1 nm, respectively, and the zeta potentials were −11.0 mV and +15.3 mV (Fig. 1(c,g)). Orange and red fluorescence derived from ZZC and R8-ZZC, respectively could be observed in a water (Fig. 1(d,h)). The fluorescence maximum wavelengths of ZZC and R8-ZZC were 657 nm and 690 nm, respectively (Fig. 1(e,i)).

### The fluorescence stability of R8-ZZC in cell culture medium

To investigate the fluorescence stability of R8-ZZC, the fluorescence images of R8-ZZC in PBS or cell culture medium under day light and UV light were observed. The red fluorescence derived from R8-ZZC could be detected for at least 7 days in PBS and cell culture medium (Fig. 1(j)). In addition, the fluorescence intensity of R8-ZZC in the cell culture medium was lower than that in PBS, however the fluorescence intensities of R8-ZZC (8 nM of ZZC) were maintained in PBS and cell culture medium (Fig. 1(k)). These data suggest that R8-ZZC can be used for cell labeling in cell culture medium.

### The optimal concentration ratio of ZZC and R8

**Figure 2:**
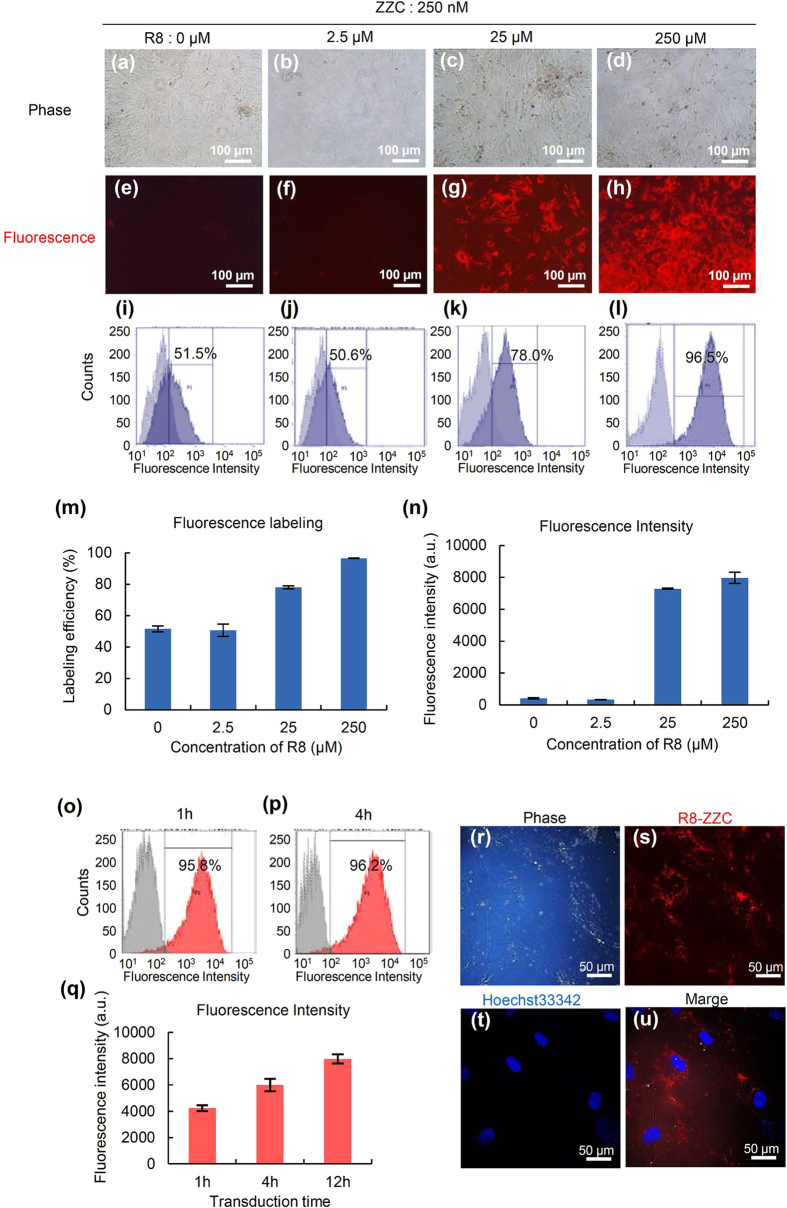
The optimal concentration ratio of ZZC and R8 for labeling ASCs. (**a–d**) The phase and (**e–h**) fluorescence images of ASCs labeled with ZZC (250 nM) and various concentrations (0, 2.5, 25, 250 μM) of R8 complexes. (**i–l**) A flow cytometry analysis of the labeling efficiency and fluorescence intensity. (**m**) Graphs of the labeling efficiency and (**n**) fluorescence intensity of ASCs labeled with ZZC (250 nM) and various concentrations (0, 2.5, 25, 250 μM) of R8 complexes as determined flow cytometry. (**o–q**) A flow cytometry analysis of the labeling efficiency and fluorescence intensity of ASCs labeled with the optimal concentration ratio of ZZC (250 nM) and R8 (250 μM) under various transduction times. (**r–u**) Confocal microscopy images of R8-ZZC-labeled ASCs ((**r**) phase contrast of ASCs, (**s**) the red fluorescence of ZZC, (**t**) the blue fluorescence of a nucleus stained with Hoechst33342, and (**u**) a merged image).

To investigate the optimal concentration ratio of ZZC and R8 for the labeling of ASCs, 250 nM of ZZC was mixed with various concentrations of R8 (0, 2.5, 25 and 250 μM), and these complexes were transduced into ASCs for 12 h. The red fluorescence of ZZC-labeled ASCs could be detected when the R8 concentration of more than 25 μM were used (Fig. 2(a–h)). The labeling efficiency and fluorescence intensity of R8-ZZC for ASCs were 78.0% and 7290 (25 μM R8), and 96.5% and 7973 (250 μM R8), respectively (Fig. 2(i–n)).

In addition, to decide the optimal transduction time of R8-ZZC to ASCs, R8-ZZC (ZZC:R8 = 1:1000) was transduced into ASCs for 1, 4 and 12 h, and then the labeling efficiency and intensity were assessed. The labeling efficiency and the fluorescence intensity were 95.8% and 4235 (1 h); 96.2% and 6006 (4 h); and 96.5% and 7973 (12 h), respectively. Notably, more than 95% of the ASCs could be labeled within 1 h. (Fig. 2(o–q)). The transduction of ZZC using R8 into ASCs was also observed as red fluorescence derived from ZZC by high-speed multiphoton confocal laser microscopy (Fig. 2(r–u)). These data suggest that the optimal concentration ratio of ZZC to R8 is 1:1000, and that almost all ASCs could be labeled with R8-ZZC by at least 1 h of transduction.

### The cytotoxicity of R8-ZZC to ASCs

**Figure 3:**
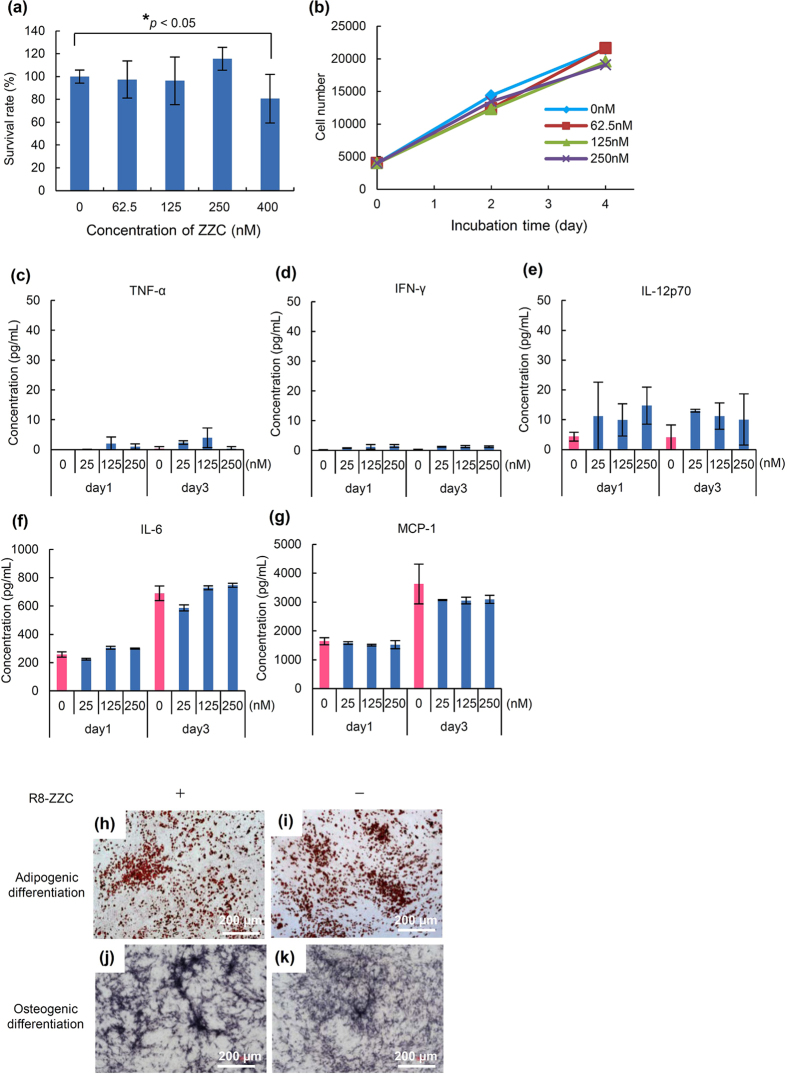
The influence of R8-ZZC on ASCs. (**a**) The cytotoxicity of R8-ZZC (0, 62.5, 125, 250, 400 nM of ZZC) on ASCs at 24 h incubation after 4 h transduction, and (**b**) the proliferation rate of R8-ZZC-labeled ASCs (0, 62.5, 125, 250 nM of ZZC) after 4 h transduction. The data are shown as the mean ± SD. *p < 0.05. (**c–g**) The major inflammatory cytokine levels (TNF-α, IFN-γ, IL-12p70, IL-6 and MCP-1) of R8-ZZC-labeled ASCs (0, 62.5, 125, 250 nM of ZZC) after 1 or 3 days of incubation. (**h,j**) The differentiation of R8-ZZC-labeled ASCs (250 nM of ZZC) or (**i,k**) non-labeled ASCs into adipocytes and osteocytes.

To examine the cytotoxicity of R8-ZZC to ASCs, various concentrations (0, 62.5, 125, 250 and 400 nM) of ZZC were transduced into ASCs using R8 for 4 h at the optimal ratio, and the ASCs were incubated for 24 h. More than 95% of the ASCs were confirmed to be alive in the ZZC concentration of less than or equal to 250 nM. Significant cytotoxicity was observed in the ASCs transduced with 400 nM of ZZC, however, more than 80% of the cells remained alive (Fig. 3(a)).

The influence R8-ZZC on the proliferation rate of ASCs was also examined within the non-cytotoxic range of concentrations. ASCs were confirmed to exhibit a growth rate that was nearly equal to normal ASCs. There were no significant differences in these concentrations (Fig. 3(b)). These data suggest that ASCs can be labeled in a ZZC concentration of less than or equal to 250 nM using R8.

### The inflammatory cytokine production of R8-ZZC-labeled ASCs

To investigate whether R8-ZZC labeling causes an inflammation reaction in ASCs, the levels of major inflammatory cytokines were measured in the culture supernatant of R8-ZZC-labeled ASCs (250 nM of ZZC) after 1 or 3 days of culturing and the production level was compared with that in non-labeled ASCs. No significant differences were observed between the non-labeled and labeled ASCs in the levels of the major inflammation cytokines (TNF-α, IFN-γ, IL-12p70, IL-6 and MCP-1). The level of IL-12p70 tended to be higher in R8-ZZC-labeled ASCs in comparison to non-labeled ASCs, however, the differences was not statistically significant. No significant differences were observed in the levels of the other cytokines. In addition, concentration-dependent changes were observed with different concentrations of R8-ZZC (Fig. 3(c–g)). These data suggest that ZZC does not influence the inflammation of ASCs.

### The differentiation of R8-ZZC-labeled ASCs

To examine the influence of R8-ZZC (250 nM of ZZC) on the differentiation capacity of ASCs, normal (non-labeled) and labeled ASCs transduced with R8-ZZC were differentiated into adipocytes and osteoblasts. The differentiations of R8-ZZC-labeled ASCs into both adipocytes and osteoblasts was similar to that observed in normal ASCs (Fig. 3(h–k)). These data suggest that R8-ZZC (250 nM of ZZC) does not affect the differentiation of ASCs.

### *In vitro* fluorescence imaging of transplanted R8-ZZC-labeled ASCs

**Figure 4:**
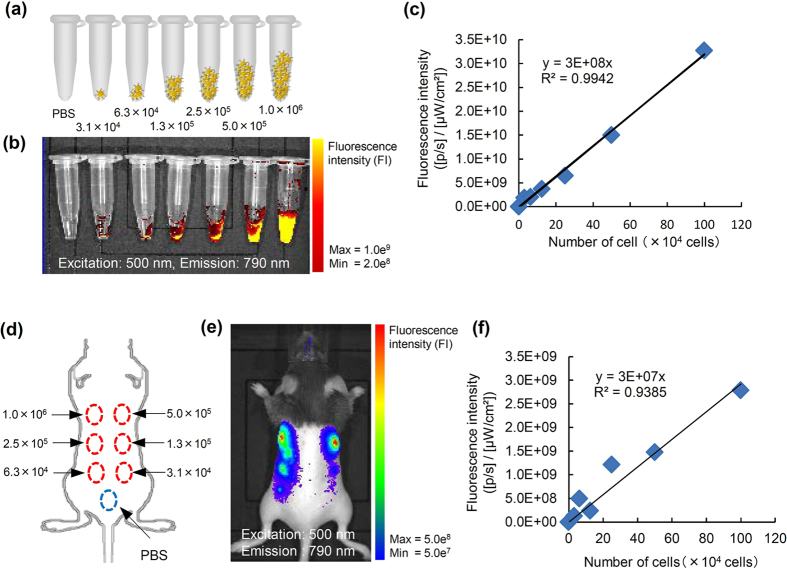
*In vivo* fluorescence imaging of the mouse R8-ZZC-labeled ASCs after subcutaneous transplantation. (**a,b**) *In vitro* fluorescence imaging of various numbers of ASCs (3.1 × 10^4^, 6.3 × 10^4^, 1.3 × 10^5^, 2.5 × 10^5^, 5.0 × 10^5^, 1.0 × 10^6^ cells) labeled with R8-ZZC (250 nM of ZZC). Excitation: 500 nm, Emission: 790 nm. (**c**) The linear relationship between the number of R8-ZZC-labeled ASCs and the fluorescence intensity (R^2^ = 0.9942). (**d,e**) *In vivo* fluorescence imaging of ASCs (3.1 × 10^4^, 6.3 × 10^4^, 1.3 × 10^5^, 2.5 × 10^5^, 5.0 × 10^5^, 1.0 × 10^6^ cells) labeled with R8-ZZC (250 nM of ZZC) after subcutaneous transplantation onto the back of a mouse. Excitation: 500 nm, Emission: 790 nm. (**f**) The linear relationship between the number of R8-ZZC-labeled ASCs and the fluorescence intensity (R^2^ = 0.9385).

To examine whether R8-ZZC-labeled ASCs (250 nM of ZZC) could be quantitatively detected, various numbers of the labeled ASCs (3.1 × 10^4^, 6.3 × 10^4^, 1.3 × 10^5^, 2.5 × 10^5^, 5.0 × 10^5^, 1.0 × 10^6^ cells) were collected in PBS and spun down. The cell pellets were then prepared for a fluorescence analysis in microtubes (Fig. 4(a)). The labeled cell pellets could be quantitatively detected at high levels of fluorescence intensity (Fig. 4(b,c)). These data suggest that the R8-ZZC-labeled cells could be detected when the fluorescence intensity was sufficient for cell visualization by fluorescence imaging.

### *In vivo* fluorescence imaging of the transplanted R8-ZZC-labeled ASCs

**Figure 5:**
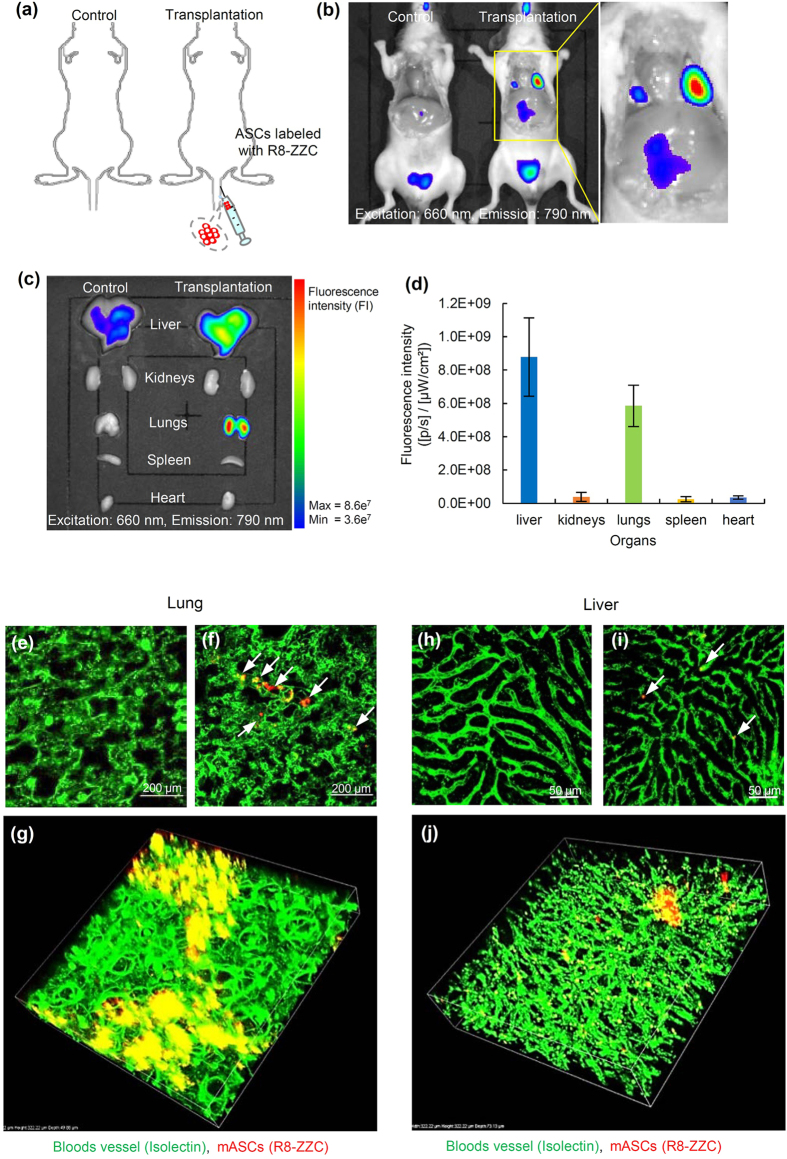
*In vivo* and *ex vivo* fluorescence imaging of R8-ZZC-labeled ASCs after intravenous transplantation. (**a,b**) *In vivo* fluorescence imaging of ASCs (1.0 × 10^6^ cells) labeled with R8-ZZC (250 nM of ZZC) after intravenous transplantation. (**c,d**) *Ex vivo* imaging and the fluorescence intensity of five major organs (liver, kidneys, lungs, spleen and heart) eviscerated from a transplanted mouse. (**e**) Multiphoton confocal images of the lung eviscerated from a control mouse (non-transplantation) and (**f**) a mouse after the transplantation of R8-ZZC-labeled ASCs (250 nM of ZZC). (**g**) A 3D confocal image of the lung eviscerated from the mouse after transplantation of R8-ZZC-labeled ASCs (250 nM of ZZC). (**h**) Multiphoton confocal images of the liver eviscerated from a control mouse (non-transplantation) and (**i**) a mouse after the transplantation of R8-ZZC-labeled ASCs (250 nM of ZZC). (**j**) A 3D confocal image of the liver from a mouse after the transplantation of R8-ZZC-labeled ASCs (250 nM of ZZC). The blood vessels and ASCs were stained with isolectin (green fluorescence) and ZZC (red fluorescence), respectively.

To assess whether images of transplanted R8-ZZC-labled ASCs (250 nM of ZZC) could be obtained in mice, various numbers of the labeled ASCs (3.1 × 10^4^, 6.3 × 10^4^, 1.3 × 10^5^, 2.5 × 10^5^, 5.0 × 10^5^, 1.0 × 10^6^ cells) were transplanted on the backs of mice (Fig. 4(d)). The transplanted R8-ZZC-labeled ASCs could be detected quantitatively as well as by *in vitro* fluorescence imaging (Fig. 4(e,f)). Moreover, R8-ZZC-labeled ASCs (1.0 × 10^6^ cells) were transplanted through the tail vein of a mouse to examine whether the fluorescence derived from ZZC could be detected and the distribution of the transplanted ASCs could be shown in the major five organs (Fig. 5(a)). When mice were sacrificed at 10 min after transplantation, the fluorescence derived from ZZC could be detected in the lungs and liver using an IVIS imaging system (Fig. 5(b)).

### *Ex vivo* fluorescence imaging of the transplanted R8-ZZC-labeled ASCs

Five organs (liver, kidneys, lungs, spleen and heart), in which almost all of the transplanted ASCs were predicted to accumulate, were isolated from mice transplanted with R8-ZZC-labeled ASCs, and *ex vivo* fluorescence imaging was conducted. The strong fluorescence derived from ZZC was detected from the lungs and liver, little or no fluorescence was detected from the kidneys, spleen and heart (Fig. 5(c)). The fluorescence intensity of *ex vivo* fluorescence imaging is closely reflected, the level of ZZC maintained inside each organ, because of the deep penetration of the excitation and the emission light in the NIR region. Consequently, the rates of transplanted ASCs than accumulated in the lungs and liver were approximately 40% and 60%, respectively (Fig. 5(d)).

Moreover, to investigate the accumulation condition in which transplanted R8-ZZC-labeled ASCs in the lungs and liver, isolated lungs and liver in which the vessels showed with green fluorescence due to isolectin staining were observed under high-speed multiphoton confocal laser microscopy. Red fluorescence derived from the transplanted R8-ZZC-labeled ASCs was observed in both the lungs and liver, however the accumulation was in two types of organs (Fig. 5(e,f,h,i)). ASCs were observed to aggregate with high efficiency in the lungs, whereas almost all of the ASCs isolated and few aggregates were observed in the blood vessels of the liver (Fig. 5(g,j)). These data suggest that ZZC is useful for the *in vivo* visualization of transplanted ASCs.

## Discussion

We described the successful synthesis of water soluble ZnS-ZAIS with carboxyl group (ZZC) nanoparticles that show fluorescence at approximately 650 nm, almost all of the particles were smaller than 10 nm and were negatively charged. ZZC could show the strong orange fluorescence in water solution. On the other hand, R8-ZZC was positively charged due to the presence of R8 molecules which is a famous cell-penetrating peptide, and could show strong red fluorescence in water solution. The fluorescence maximum wavelength of R8-ZZC was confirmed to shift to about 20 nm longer wavelength in comparison to ZZC. This fluorescence color shift to a slightly longer wavelength by the attachment of R8 molecules may have been caused by a small change in size (from 5.1 nm to 9.1 nm). X. Gao *et al*. showed that the addition of fibrinogen (fib) to the CuInS_2_ QDs solution led to the formation of a Fib–CuInS_2_ QDs complex through electrostatic interactions, and resulting in a red shift of the peak of fluorescence wavelength^28^. R8-ZZC maintained its strong red fluorescence in PBS or cell culture medium for at least 1 week. R8-ZZC was therefore suitable for cell labeling in cell culture medium.

The optimal complex ratio of ZZC and R8 molecules for ASCs labeling was 1:1000. We have already revealed that the optimal complex ratio CdSe-based QDs (Invitrogen; Qdot ITK Carboxyl Quantum Dots) and R8 molecules is 1:10000,^14^ thus it required one-tenth the number of R8 molecules that were needed for ZZC. It is thought that this is due to the size of the nanoparticles and the amount of carboxyl group on the surface. On the other hand, the labeling time was not changed, and almost all of the ASCs could be labeled with R8-ZZC within 1 h. Cell-penetrating peptides, including R8 molecules, are reported to be introduced into cells via macropinocytosis in the endocytosis pathway^29^,^30^. Thus R8-ZZC is also thought to be introduced into ASCs by macropinocytosis. R8-ZZC is therefore expected to have the potential to quickly label many types of cells, including stem cells.

The cytotoxicity of CdSe-based QDs (Invitrogen; Qdot ITK Carboxyl Quantum Dots) to ASCs was revealed greater than or equal to that observed in QDs at a concentration of 16 nM, however, R8-ZZC was confirmed to have no cytotoxic effects in ASCs at ZZC concentrations less than or equal to 250 nM^14^. ZZC does not contain cytotoxic elements (such as Cd and Se), thus the cytotoxicity of R8-ZZC to ASCs appears to be extremely suppressed in comparison to CdSe-based QDs^18^. The main cause appears to be due to the absence of toxicity elements. In addition, R8-ZZC provided a rounded shape in comparison to CdSe-based QDs having a triangular shape. Thus, the rounded shape of R8-ZZC is considered to contribute to reduce the cytotoxicity to ASCs. No influence of R8-ZZC on the self-renewal or the ability to differentiate into adipocytes or osteocytes was confirmed at ZZC concentrations less than or equal to 250 nM.

The influence of influence of R8-ZZC on the production of inflammatory cytokines in ASCs was also examined. Ho *et al*. reported that CdSe-based QDs induced MCP-1 expression via the MyD88-dependent Toll-like receptor signaling pathways in macrophages^31^. Furthermore, Wang *et al*. assessed the immunotoxicity of CdSe-based QDs in macrophages, lymphocytes and BALB/c mice^32^. However, little is known about the influence of various types of QDs on the production of inflammatory cytokines in ASCs. In the present study, the levels of MCP-1 and IL-6 was found to be increased by labeling with 8 nM of CdSe-based QDs (data not shown). In contrast, R8-ZZC did not induce the production of major inflammatory cytokines. These results may be the first report of the influence of QDs on the inflammatory cytokines in ASCs. Based on the finding of the present study, R8-ZZC is expected to be more useful for stem cell labeling than CdSe-based QDs. In the future, however, further investigations should be performed to elucidate the detailed mechanisms underlying the differences in the cytotoxicity of CdSe-based QDs and ZZC.

R8-ZZC-labeled ASCs in the microtube could be quantitatively detected (depending on the number of ASCs) by analyzing the fluorescence intensity using an *in vivo* fluorescence imaging system. The subcutaneously transplanted R8-ZZC-labaled ASCs on the backs of mice could also be quantitatively detected. In addition, R8-ZZC-labeled ASCs that were intravenously transplanted through the tail vein could be detected, and similarly to previous reports) almost all of the transplanted ASCs were found to accumulate in the lungs and liver. However, the details of the accumulation of transplanted ASCs in the lungs and liver have remained poorly understood. We therefore investigated the accumulation of transplanted ASCs using high-speed multiphoton confocal laser microscopy and found that the conditions in the lungs were different to those in the liver.

The transplanted ASCs formed aggregates at many sites in the lungs, but not in the liver. The blood vessels in the lungs were observed to be very fine (Fig. 5(g)). It was therefore hypothesize that the size of the transplanted ASCs might have caused them to aggregate at the same point in a narrow vein. In contrast, the blood vessels in the liver were not as fine (Fig. 5(j)), thus individually ASCs appeared to accumulate over relatively large areas of the liver. However, almost all of the ASCs were found in the blood vessels, and they could not pass the perisinusoidal space. If the transplanted ASCs are maintained in the liver for a long period of time and differentiate into hepatocytes with high efficiency, then a sinusoidal dilator may be useful. This study suggests that ZZC labeling using R8 can be used for the labeling and *in vivo* imaging of ASCs. The findings of the present study are expected to contribute to further progress in their clinical applications.

## Conclusion

The safety of newly synthesized form of aqueous carboxylated ZnS-ZAIS nanoparticles (ZZC) was assessed. ZZC showed strong fluorescence in cell culture medium, and a complex of ZZC and R8 peptides (R8-ZZC) was able to label ASCs with high efficiency. The cytotoxicity of R8-ZZC to ASCs was found to be extremely low in comparison to CdSe-based QDs, and R8-ZZC was not found to influence the proliferation rate or differentiation ability of ASCs. In addition, R8-ZZC did not induce the production of major inflammation cytokines (TNF-α, IFN-γ, IL-12p70, IL-6 and MCP-1) in ASCs. The transplanted R8-ZZC-labeled ASCs could be quantitatively detected in the lungs and liver mainly using an *in vivo* imaging system. The transplanted ASCs were found to aggregate at many sites in the lungs, whereas they were found to individually accumulate in the liver using high-speed multiphoton confocal laser microscopy. These findings suggest that R8-ZZC could be utilized for the labeling and *in vivo* visualization of transplanted ASCs.

## Methods

### Materials

Qdot ITK Carboxyl Quantum dots (QDs), Hank’s balanced salt solution and ELISA Kits for mouse TNF-α, IL-1β, IL-6 and VEGF were purchased from Life Technologies ^TM^ Japan (Tokyo, Japan). Fetal bovine serum (FBS) was purchased from Trace Scientific Ltd. (Melbourne, Australia). Octa-arginine peptide (R8) was synthesized and purchased from Sigma Aldrich Japan (Tokyo, Japan). Collagenase Type II was purchased from Koken Co., Ltd. (Tokyo, Japan). Novo-Heparin (10,000 Units/10 mL for injection) was purchased from Mochida Pharmaceutical Co., Ltd. (Tokyo, Japan). A cytometric bead array (CBA) mouse inflammation kit was purchased from Japan BD Bioscience (Tokyo, Japan). Non-luminescence feed without alfalfa was purchased from SLC Japan, Inc. (Tokyo, Japan). A cell counting kit-8 (CCK-8) was purchased from DOJINDO Laboratories (Kumamoto, Japan). GSL I-B4 Isolectin FITC conjugated was purchased from Funakoshi Co., Ltd. (Tokyo, Japan).

### Animals

C57BL/6 mice were purchased from Japan SLC (Hamamatsu, Japan). The mice were housed in a controlled environment (12 h light/dark cycles at 22 °C) with *ad libitum* access to water and a standard chow diet before they were killed. The conditions and the handling of the animals in this study were approved by the Nagoya University Committee on Animal Use and Care and the experiments were carried out in accordance with the Regulations on Animal Experimentation at Nagoya University.

### The isolation and culture of ASCs

The isolation and culture of ASCs have been reported previously. Briefly, 7- to 14-month-old female C57BL/6 mice were killed by cervical dislocation, and adipose tissue specimens were isolated from the inguinal groove and washed by Hank’s buffer to remove the blood cells. The adipose tissues were cut finely and digested with type II collagenase at 37 °C in a shaking water bath for 90 min. Adipose tissue cells were suspended in the cell culture medium (Dulbecco’s modified Eagle’s medium (DMEM)/F12 containing 20% FBS and 100 U/mL penicillin/streptomycin). The cells were centrifuged at 1,200 rpm for 5 min at room temperature to obtain pellets containing the ASCs. The cells were washed three times by suspension and centrifugation in the culture medium. The primary cells were cultured for 4–5 days until they reached confluence and were defined as passage “0”. Cells from passages 2–5 were used in all of the experiments.

### The synthesis of oleylamine-modified ZnS-AgInS_2_ solid solution nanoparticles (ZAIS)

Oleylamine-covered ZAIS nanoparticles were prepared according to the procedure paper with a slight modification. Ag(S_2_CNEt_2_) (13.6 mg), In(S_2_CNEt_2_)_3_ (29.7 mg), and Zn(S_2_CNEt_2_)_2_ (6.8 mg) were dispersed in oleylamine (3.0 mL) and the reaction solution was heated to 180 °C for 30 min under an N_2_ atmosphere^22^. The resulting suspension was centrifuged at 4000 rpm for 5 min to remove large particles. ZAIS nanoparticles were separated from the supernatant with the addition of methanol, followed by centrifugation for 5 min at 4000 rpm.

### The synthesis of ZnS-coated ZAIS nanoparticles (ZnS-ZAIS)

ZnS-ZAIS nanoparticles were prepared according to a previously published procedure^23^. ZAIS nanoparticles, which were prepared as described above, zinc acetate dihydrate (11.8 mg), and thioacetamide (4.0 mg) were dissolved in oleylamine (2.0 mL) and the reaction solution was heat-treated at 180 °C for 30 min under an N_2_ atmosphere. After filtration through a syringe filter, the nanoparticles that were obtained were separated from the solution by the addition of methanol, followed by centrifugation for 5 min at 4000 rpm.

### The synthesis of ZnS-ZAIS modified with carboxyl group (ZnS-ZAIS-COOH:ZZC)

ZnS-ZAIS-COOH was prepared by a procedure similar to that described paper^33^. ZnS-ZAIS nanoparticles, which were prepared above, were dissolved in chloroform (1.0 mL). Methanol (1.0 mL) solution containing sodium 3-mercaptopropionic acid (MPA) (100 μL) and tetramethylammonium hydride 25% methanol solution (730 μL) was mixed with the ZnS-ZAIS nanoparticle solution, and the reaction solution was heated to 70 °C for approximately 4 h under an N_2_ atmosphere. After the solvent was removed under reduced pressure, the crude product was dissolved in methanol. Chloroform was then added to precipitate ZnS-ZAIS-COOH. Finally, centrifugation was performed for 5 min at 4000 rpm. This purification process was repeated several times to remove residual reagents. The resulting precipitate was dried under a vacuum and dissolved in MilliQ water.

### The particle properties

The particle size distribution and the zeta potential of ZZC and the R8-ZZC complex (R8-ZZC) in water were measured using a dynamic light-scattering spectrophotometer (ZETASIZER Nano-ZS, Malvern Instruments Limited Japan, Hyogo, Japan). The particle size is shown by the peak of the size distribution graph in Fig. 1(b–i).

The absorbance spectra of ZZC and R8-ZZC were measured using an 8453 A UV-visible spectrophotometer (Agilent Technology, Santa Clara, CA, USA). The fluorescence spectra were measured using a photonic multichannel analyzer (PMA-12; Hamamatsu Photonics, Shizuoka, Japan). The excitation wavelength was 365 nm. Transmission electron microscopy (TEM, H-7650, Hitachi, Japan) was used to visualize the ZZC and R8-ZZC nanoparticles at an accelerating voltage of 100 kV.

### The fluorescence stability of R8-ZZC in PBS and cell culture medium

R8-ZZC (ZZC:250 nM, R8:250 μM) in PBS or transduction medium (DMEM/F12 containing 2% FBS and 100 U/mL penicillin/streptomycin) was incubated for various length of time (0, 1, 3, 5 and 7 days) at 37 °C. The phase and fluorescence images were observed by 8 mega pixel digital camera and the intensities were measured using an IVIS Lumina K Series III (PerkinElmer, Inc., Massachusetts, USA).

### The transduction of R8-ZZC into ASCs

ZZC (250 nM) and R8 were mixed for 15 min at room temperature in various ratios (ZZC:R8 = 1:0, 1:10, 1:100, 1:1000) in order to determine the optimal concentration ratio of ZZC/R8 for inducing transduction into ASCs. ASCs were incubated with R8-ZZC in a transduction medium at 37 °C. After 4 h incubation, the cells were washed using transduction medium. The transduction of ZZC into ASCs was the observed by phase-contrast fluorescence microscopy and high-speed multiphoton confocal laser microscopy (A1MP^+^/A1RMP^+^, Nikon, Tokyo, Japan). The transduction efficiency was evaluated by flow cytometry (BD LSRFortessa™ X-20, Japan BD, Tokyo, Japan).

### The cytotoxicity of ZZC to ASCs

ASCs (1 × 10^4^ cells) were seeded in a 96-well plate (BD Falcon; BD Biosciences,) with 100 μL of culture medium, and the cells were cultured for 24 h. ZZC (0–400 nM) and R8 were mixed at the optimal ratio of 1:1000, and R8-ZZC nanoparticles were transduced into ASCs in a transduction medium. After 4 h transduction, the ASCs were washed twice with a transduction medium, and were incubated for 24 h. The viable cells were then counted using a cell counting kit-8 (CCK-8). CCK-8 reagent (10 μL) was added to each well and the reaction was allowed to proceed for 2 h. The absorbance of the sample at 450 nm was measured against a background control using a microplate reader (POLARstar OPTIMA; BMG LABTECH, Ortenberg, Germany).

### The proliferation of R8-ZZC-labeled ASCs

ASCs (4 × 10^3^ cells) were seeded in a 96-well plate with 100 μL of culture medium for 1 day and then transduced with ZZC using R8 at various concentrations for 4 h. ZZC (0, 62.5, 125, 250 nM) and R8 were mixed at the optimal ratio of 1:1000, and then each complex was transduced into ASCs. The cells were the washed and the medium was replaced with 100 μL of new culture medium. After 2 and 4 days, the viable cells were counted using a CCK-8. The method was the same as that which was used to determine cytotoxicity.

### Adipogenic and osteogenic differentiation

Adipogenic differentiation was induced by culturing the cells for 3 days in DMEM (high glucose) containing 100 mM indometacin, 1 mM dexamethasone, 1 mM hydrocortisone, 10 mM insulin (Sigma, I-5500) and 10% FBS. The cells were then further cultured in DMEM (high glucose) containing 10% FBS for 2 weeks and the medium was changed every 3 days. Differentiation was confirmed by the conventional microscopic observation of intracellular lipid droplets. Oil Red O staining was used as an indicator of intracellular lipid accumulation.

Osteogenic differentiation was induced by culturing the cells for 2 weeks in DMEM containing 200 mM dexamethasone, 50 mM ascorbate-2-phosphate (Wako Pure Chemical Industries Ltd., 013-12061), 10 mM a-glycerophosphate (Sigma, G-9891) and 10% FBS. The differentiation was confirmed by the staining of any alkaline phosphatase activity.

### The measurement of inflammation markers

ASCs (1 × 10^5^ cells) were seeded into a 24-well plate and cultured for 24 h. ZZC (0, 25, 125, 250 nM) and R8 were mixed at the optimal ratio of 1:1000, and each complex was transduced into ASCs. After 4 h incubation, the ASCs were replaced with new cell culture medium and were cultured for 1 or 3 days. The cell culture medium was collected and the levels of five types of cytokines TNF-α, IFN-γ, IL-12p70, IL-6 and MCP-1) were measured by using Cytometric Bead Array Mouse Inflammation Kit (BD Bioscience, 552364).

### *In vivo* and *ex vivo* fluorescence imaging of R8-ZZC-labeled ASCs

ZZC was transduced into ASCs using R8 in the same manner as described above. The fluorescence images and intensities of ASCs (0.31, 0.63, 1.3, 2.5, 5.0, and 10 × 10^5^ cells) labeled with ZZC (250 nM) in 1.5 mL tubes were investigate using an IVIS Lumina K Series III (excitation filter: 500 nm ± 20 nm, emission filter: 790 nm ± 40 nm). Next, the R8-ZZC-labeled ASCs were subcutaneously transplanted with PBS (50 μL) into the backs of C57BL/6 mice. The fluorescence images and intensities were investigated using an IVIS Lumina K Series III with the above-described filter conditions. The correlation coefficient between the number of R8-ZZC-labeled ASCs and the fluorescence intensity was calculated.

For the *in vivo* fluorescence imaging studies, we used mice that were given food which did not include any fluorescence components (alfalfa-free feed) for 1 week in order to reduce the effects of the autogenic fluorescence from the gastrointestinal tract. R8-ZZC-labeled ASCs (1 × 10^6^ cells) were then transplanted with saline (0.15 mL) in combination with heparin through the tail vein. The mice were anesthetized and were monitored from 10 min after transplantation using an IVIS Lumina K Series III (excitation filter: 660 nm ± 20 nm), emission filter: 790 nm ± 40 nm). Next, for *ex vivo* fluorescence imaging, the major organs (liver, kidneys, lungs, spleen and heart) were harvested and immediately subjected to fluorescence imaging using an IVIS Lumina K Series III with the same conditions that were used for *in vivo* imaging. The region of intensity (ROI) was measured with the assistance of the IVIS Lumina K Series III.

### Observation of the R8-ZZC-labeled ASCs in the organs

Mice transplanted with R8-ZZc-labeled ASCs were sacrificed, and then the five major organs (heart, lungs, liver, spleen and kidneys) were extracted. These organs were dipped in PBS (500 μL) with isolectin conjugated with FITC (5 μL) for 12 h in order to dye and clear the blood vessels in the organs. The organs were then placed on a 35 φmm glass bottom dish and observed under high-speed multiphoton confocal laser microscopy (A1MP^+^/A1RMP^+^, Nikon, Tokyo, Japan).

### Statistical analysis

The numerical values are presented as the mean ± SD. Each experiment was repeated three times. Statistical significance was evaluated using an unpaired Student’s *t*-test for comparisons between two groups; *P*-values of < 0.05 were considered to indicate statistically significance. All of the statistical analyses were performed using the SPSS software package.

## Additional Information

**How to cite this article**: Ogihara, Y. *et al*. Labeling and *in vivo* visualization of transplanted adipose tissue-derived stem cells with safe cadmium-free aqueous ZnS coating of ZnS-AgInS_2_ nanoparticles. *Sci. Rep.* **7**, 40047; doi: 10.1038/srep40047 (2017).

**Publisher's note:** Springer Nature remains neutral with regard to jurisdictional claims in published maps and institutional affiliations.
